# Based on systematic druggable genome-wide Mendelian randomization identifies therapeutic targets for diabetes

**DOI:** 10.3389/fendo.2024.1366290

**Published:** 2024-06-10

**Authors:** Hu Li, Wei Li, Dongyang Li, Lijuan Yuan, Yucheng Xu, Pengtao Su, Liqiang Wu, Zhiqiang Zhang

**Affiliations:** ^1^ Emergency Department, Binzhou Medical University Hospital, Binzhou, China; ^2^ Urology Department, Affiliated Hospital of Guizhou Medical University, Guiyang, China; ^3^ Internal Medicine-Neurology, Binzhou Medical University Hospital, Binzhou, China; ^4^ Department of Critical Care Medicine, Jinan Central Hospital, Jinan, China

**Keywords:** diabetes, drug targets, genetics research, Mendelian randomization, genetic effect analysis

## Abstract

**Purpose:**

Diabetes and its complications cause a heavy burden of disease worldwide. In recent years, Mendelian randomization (MR) has been widely used to discover the pathogenesis and epidemiology of diseases, as well as to discover new therapeutic targets. Therefore, based on systematic “druggable” genomics, we aim to identify new therapeutic targets for diabetes and analyze its pathophysiological mechanisms to promote its new therapeutic strategies.

**Material and method:**

We used double sample MR to integrate the identified druggable genomics to evaluate the causal effect of quantitative trait loci (eQTLs) expressed by druggable genes in blood on type 1 and 2 diabetes (T1DM and T2DM). Repeat the study using different data sources on diabetes and its complications to verify the identified genes. Not only that, we also use Bayesian co-localization analysis to evaluate the posterior probabilities of different causal variations, shared causal variations, and co-localization probabilities to examine the possibility of genetic confounding. Finally, using diabetes markers with available genome-wide association studies data, we evaluated the causal relationship between established diabetes markers to explore possible mechanisms.

**Result:**

Overall, a total of 4,477 unique druggable genes have been gathered. After filtering using methods such as Bonferroni significance (P<1.90e-05), the MR Steiger directionality test, Bayesian co-localization analysis, and validation with different datasets, Finally, 7 potential druggable genes that may affect the results of T1DM and 7 potential druggable genes that may affect the results of T2DM were identified. Reverse MR suggests that C4B may play a bidirectional role in the pathogenesis of T1DM, and none of the other 13 target genes have a reverse causal relationship. And the 7 target genes in T2DM may each affect the biomarkers of T2DM to mediate the pathogenesis of T2DM.

**Conclusion:**

This study provides genetic evidence supporting the potential therapeutic benefits of targeting seven druggable genes, namely MAP3K13, KCNJ11, REG4, KIF11, CCNE2, PEAK1, and NRBP1, for T2DM treatment. Similarly, targeting seven druggable genes, namely ERBB3, C4B, CD69, PTPN22, IL27, ATP2A1, and LT-β, has The potential therapeutic benefits of T1DM treatment. This will provide new ideas for the treatment of diabetes and also help to determine the priority of drug development for diabetes.

## Background

Diabetes is a chronic metabolic disorder that is characterized by an increase in blood sugar levels caused by absolute or relative insulin deficiency (1). It is estimated that one in every 10 people in the world has diabetes, and this number will continue to increase in the coming decades. The International Diabetes Federation estimates that the number of diabetes patients is expected to increase from 425 million adults in 2017 to 629 million in 2045. This will affect approximately 9.9% of the global population, resulting in an increasingly unsustainable global health burden (2, 3). Moreover, diabetes complications also lead to a heavy burden of disease worldwide. Acute complications of diabetes (such as diabetes ketoacidosis [DKA], hypertonic hyperglycemic coma [HHS]) and vascular complications of diabetes macrovascular and microvascular systems (such as cardiovascular disease [CVD], diabetes nephropathy [DKD], diabetes retinopathy [DR], neuropathy, and diabetes foot) are the main causes of quality of life impairment and death in diabetes patients, which has brought a serious burden to global public health (4, 5).

However, the current treatment of diabetes often relies on two main ways: changing lifestyles (such as diet, exercise, and weight loss) and lifelong use of hypoglycemic drugs. Since the 1950s, eight types of hypoglycemic drugs have been approved and widely used by the global Food and Drug Administration. This includes insulin preparations, traditional oral hypoglycemic drugs (metformin, thiazolidinediones (TZDs), sulfonylureas (SUs), glinides, and α-glucosidase inhibitors), modern therapies based on intestinal proinsulin (such as dipeptidyl peptidase-4 inhibitors (DPP4is) and glucagon-like peptide-1 receptor agonists (GLP-1RA), and sodium glucose cotransporter-2 inhibitors (SGLT2is) (6, 7). However, in the case of traditional hypoglycemic drugs, their adverse events (hypoglycemia, weight gain, gastrointestinal disorders, psychological insulin resistance, and edema) can negatively affect the acceptability of treatment, thereby reducing patient satisfaction and adherence to the therapy (8). Compared with traditional oral drugs, incretin-based therapies (DPP4is and GLP-1RA) and SGLT2is are new drugs for the treatment of diabetes. They play an important role in delaying the complications of diabetes and controlling weight (9). SGLT2is (engagliflozin and dapagliflozin) has a strong role in reducing the risk of hospitalization for heart failure, slowing down the progression of diabetes nephropathy, and protecting the kidney. However, due to the mechanism of action of SGLT2is (promoting urination and sodium excretion by inhibiting glucose reabsorption in proximal renal tubules), it cannot be used in patients with renal insufficiency. Meanwhile, SGLT2is can lead to accelerated loss of bone mineral density, an increased risk of fractures, and an increased risk of urinary and reproductive system infections (10, 11). DPP4is (Sigliptin) and GLP-1RA (Lilalutide) are incretin-based therapies that are helpful to control and reduce the weight of diabetes patients in the treatment of diabetes, but their side effects include severe nausea, vomiting, dizziness, and gastrointestinal discomfort, which make some patients intolerable (12). In summary, both traditional drugs that focus on insulin secretion and insulin sensitization, as well as modern therapies based on incretin-based therapies, can have unnecessary side effects on patients, leading to non-compliance and treatment failure (13). Moreover, these treatment methods cannot reverse this process. Therefore, evaluating the common genomic basis between diabetes and diabetes complications is very important for better understanding its potential pathophysiology and possibly controlling disease progression. In the past few decades, “druggable” genes or their encoded proteins have been targeted by drugs or may be used as proteins targeting small molecules or monoclonal antibodies. The “druggable” genes that simultaneously encode protein or gene expression can serve as drug targets and provide strong clues for disease treatment (14, 15).

**Figure 1:**
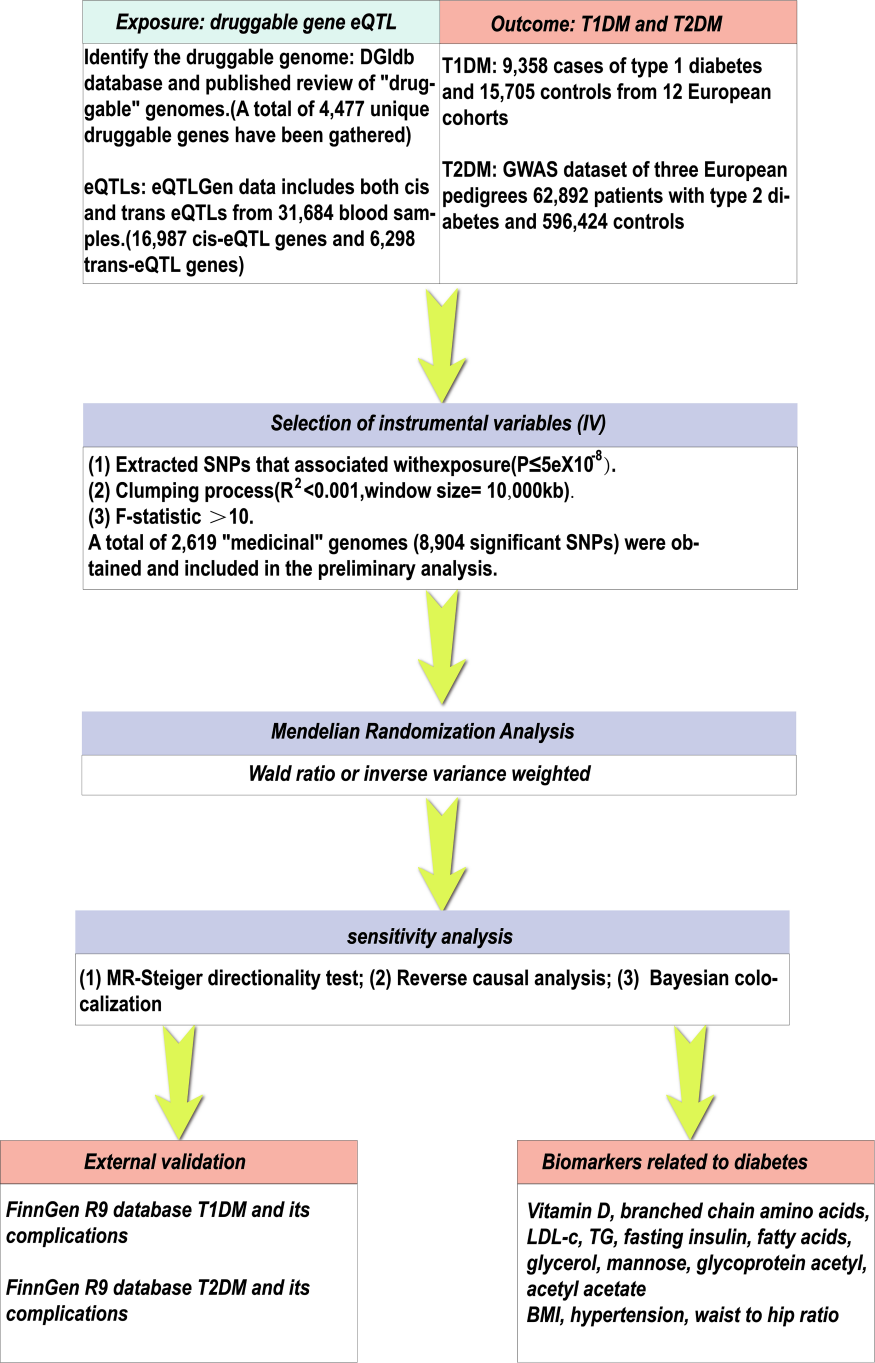
The research design and workflow of this study.

Mendelian randomization (MR) analysis provides a valuable alternative to randomized clinical trials by utilizing genetic variations associated with specific exposures. By adopting this method, the causal relationship of the disease can be evaluated and potential therapeutic targets can be identified, which can be validated in subsequent clinical trials (16). Therefore, through MR analysis, there should be many disease-specific drug targets that have not yet been developed among the thousands of other loci identified in GWAS and similar high-quality genetic associations (expression quantitative trait loci, eQTL) studies. In this study (**Figure 1**), we conducted systematic “druggable” genome-wide MR to identify therapeutic targets for diabetes. Secondly, we conducted a reverse MR analysis to assess the existence of a reverse causal relationship. Thirdly, we conducted a Steiger directionality test to eliminate SNPs in the opposite direction so as to ensure the directionality of the association between the “druggable” gene and diabetes.Fourthly, we conducted co location analysis to verify the robustness of the expression tool variable (IV), and repeated validation studies were conducted using different diabetes data sources to verify the stability of the identified druggable genes. Finally, we evaluated the causal relationship between the identified druggable genes and the established biomarkers of diabetes to explore the possible mechanisms of these genes and the pathogenesis of diabetes.

## Method

### Research design

The design of this study referred to the Mendelian Randomized Enhanced Epidemiological Observation Study Report (STROBE-MR) (17), and all participants in this study were subjects of European ancestry to reduce population stratification bias. In order to obtain the necessary data, publicly accessible datasets from eQTLGen, derived from genome-wide association studies (GWAS) and expression quantitative trait loci (eQTL) studies, were used (https://eqtlgen.org/). In addition, all the data used in this work came from studies with subject consent and ethical recognition; therefore, our study does not require ethical approval from the institutional review committee.

### Data source

#### Identification of druggable genes

Druggable genes were obtained from the Drug-Gene Interaction Database (DGIdb V.4.2.0, https://www.dgidb.org/) and a recent review on the ‘druggability’ of genes (14). The DGIdb provides information on drug-gene interactions and druggable genes from publications, databases, and other web-based sources. Obtained a total of approximately 4,477 “patent drug genes” (**Supplementary Table 1**).

#### eQTL data source

Organizational-specific eQTL data from eQTLGen (https://eqtlgen.org/). The eQTLGen data includes both cis and trans eQTLs (16,987 cis eQTLs and 6,298 trans eQTLs) from 31,684 blood samples, with the majority being healthy individuals of European ancestry (18).

#### Data source of diabetes

The data set for type 2 diabetes (T2DM) comes from a meta-analysis that combines GWAS data sets of three European pedigrees (62,892 cases of T2DM and 59,6424 controls) to determine the genetic locus of T2DM (19). The genotype data of 9,358 type 1 diabetes cases and 15,705 control subjects from 12 European cohorts were deeply interpolated in the type 1 diabetes (T1DM) dataset, and then the genome-wide association test and meta-analysis were conducted (20). All validation sets of type 1 and type 2 diabetes and its 12 complications, namely, acute complications (diabetes ketoacidosis), diabetes coma, chronic microvascular complications (diabetes retinopathy, diabetes nephropathy, and diabetes neuropathy), and chronic macrovascular complications (diabetes peripheral circulation complications), are derived from the FinnGen R9 study (https://www.finngen.fi/en/). We have listed all the data sources and detailed information in **Table 1**.

**Table 1:** The main data sources used in this study.

Study	Phenotype	Cases	Controls	PMID
eQTLGen Alliance	Genetic loci for gene (eQTL) expression in plasma	31,684	–	34475573
NA	type 2 diabetes	62,892	596,424	30054458
NA	type 1 diabetes	9,358	15,705	32005708
FinnGen R9	type 2 diabetes	38,657	310,131	NA
FinnGen R9	type 1 diabetes	8,967	308,373	NA
FinnGen R9	Type 2 diabetes with coma	4,709	308,280	NA
FinnGen R9	Type 2 diabetes with ketoacidosis	657	308,280	NA
FinnGen R9	Type 2 diabetes with neurological complications	1,894	308,280	NA
FinnGen R9	Type 2 diabetes with ophthalmic complications	4,172	308,280	NA
FinnGen R9	Type 2 diabetes with peripheral circulatory complications	2,179	308,280	NA
FinnGen R9	Type 2 diabetes with renal complications	2,684	308,280	NA
FinnGen R9	Type 1 diabetes with coma	2,050	308,280	NA
FinnGen R9	Type 1 diabetes with ketoacidosis	2,102	308,280	NA
FinnGen R9	Type 1 diabetes with neurological complications	1,077	308,280	NA
FinnGen R9	Type 1 diabetes with ophthalmic complications	5,202	308,280	NA
FinnGen R9	Type 1 diabetes with peripheral circulatory complications	669	308,280	NA
FinnGen R9	Type 1 diabetes with renal complications	1,579	308,280	NA

NA, not available.

#### Selection of IV

Select druggable genome eQTLs as exposure data. In order to generate IV, meet the assumption of MR, and obtain reliable IV, we conducted a series of strict standards on the systematic druggable genome eQTLs for screening IV: (1) Select SNPs with genome-wide significance (p<5×10–8) and an acceptable mutation probability (secondary allele frequency>1%); (2) Execute clump (r^2<0.001, kb=10,000kb) to eliminate linkage imbalance between genetic instruments; (3) The F-statistic is used to estimate the strength of each genetic instrument and select all strong instrumental variables (F > 10) (21). The formula is R^ 2×(N − 2)/(1 − R^ 2), where R^ 2 is the cumulative explained variance of selected SNPs in exposure that used (2×EAF×(1 − EAF)×beta^ 2)/[(2×EAF×(1 − EAF) ×beta^ 2) + (2×EAF×(1 − EAF)×N×SE(beta) ^ 2)], where N is the sample size of research, EAF is the effect allele frequency, beta is the estimated genetic effect, and SE(beta) is the standard error of the beta. Under strict screening conditions, a total of 2,619 “druggable” genes (8,904 significant SNPs) were included in the preliminary analysis.

#### MR analysis

In this study, we used the “druggable” gene eQTLs as exposure data and used type 1 diabetes and type 2 diabetes as outcomes data for dual-sample MR analysis. If there is only one SNP for a given gene, eQTL, use the Wald ratio. When two or more genetic instruments (SNPs) are available, inverse variance weighting (Re IVW) is the main analytical method. This method is used to combine the causal effects of individual SNPs, allowing for heterogeneity between SNPs and returning unbiased estimates of causal relationships when all IVs are valid and pleiotropy levels are balanced. For preliminary analysis, we used Bonferroni correction to adjust for multiple tests, with a threshold P-value of 0.05/2619 (P<1.90e-05) to determine the priority of the results for further analysis.

#### MR-Steiger directionality test

For the preliminary validation results, we first conducted an MR Steiger directionality test, which effectively removed SNPs with opposite causal directions. The MR-Steiger filtering hypothesis suggests that effective genetic variations should explain more exposure differences than results. If the genetic tool does not meet this criterion, the genetic variation is determined to have bidirectional effects. After removing SNPs with bidirectional effects, we perform a double-sample MR analysis again.

#### Reverse MR analysis

For the preliminary significant results obtained, we selected significant genetic instruments (SNPs) from the original exposure data of T2DM and T1DM according to the same screening criteria for “druggable” gene eQTLs and used “druggable” gene eQTL as the outcome data for bidirectional MR analysis to detect potential reverse causal relationships. The results have statistical significance at P<0.05.

#### Bayesian co-localization analysis

Bayesian co-localization analysis uses the “coloc” software package to evaluate the probability of two features sharing the same causal variable. This analysis helps to examine the possibility of genetic confounding by evaluating the posterior probabilities of different causal variations, shared causal variations, and co-localization probabilities. This analysis provides several outputs of interest. Co-localization generates a posterior probability corresponding to one of the following five assumptions: PPH0, no association with either trait; PPH1, association with the expression of the gene but not the diabetes risk; PPH2, association with the diabetes risk but not the expression of the gene; PPH3, association with the diabetes risk and expression of the gene, with distinct causal variants; and PPH4, association with the diabetes risk and expression of the gene, with a shared causal variant. We restricted our analysis to genes reaching PPH3+PPH4 of ≥0.8 owing to limited power in the co-localization analysis. We think that there is evidence of a common localization of this gene.

#### External verification

Due to differences in genetic effects between different datasets. We use the same-variant and significant-variant strategies in different datasets to replicate the primary findings and further determine the stability of our results. T2DM and its complications, as well as T1DM and its complications, are all from the FinnGen R9 study. For external validation, we still use the Bonferroni correction to adjust for multiple tests. The threshold P-value for T2DM and its complications is 0.05/13 (P<0.0038), while the threshold P-value for T1DM and its complications is 0.05/10 (P<0.005). We believe the results are significant.

#### Biomarkers related to diabetes

To determine whether the potential “druggable” gene targets identified by our MR analysis are involved in the hypothetical pathological mechanism of diabetes (mainly T2DM). We conducted another set of dual-sample MR. Among them, we used the identified potential “druggable” gene eQTL as exposure data and T2DM-related biomarkers as outcome data. To further explore the influence of “druggable” gene expression on diabetes biomarkers.

#### Statistical analysis

All MR analyses were conducted using the R (version 4.2.2) TwoSampleMR, MRPRESSO, Mendelian Randomization, and Coloc software packages. All DNA positions were constructed based on the human reference genome using hg19 (GRCh37).

## Result

### 23 potential drug targets for diabetes prevention were identified in the dual sample MR phase

**Figure 2:**
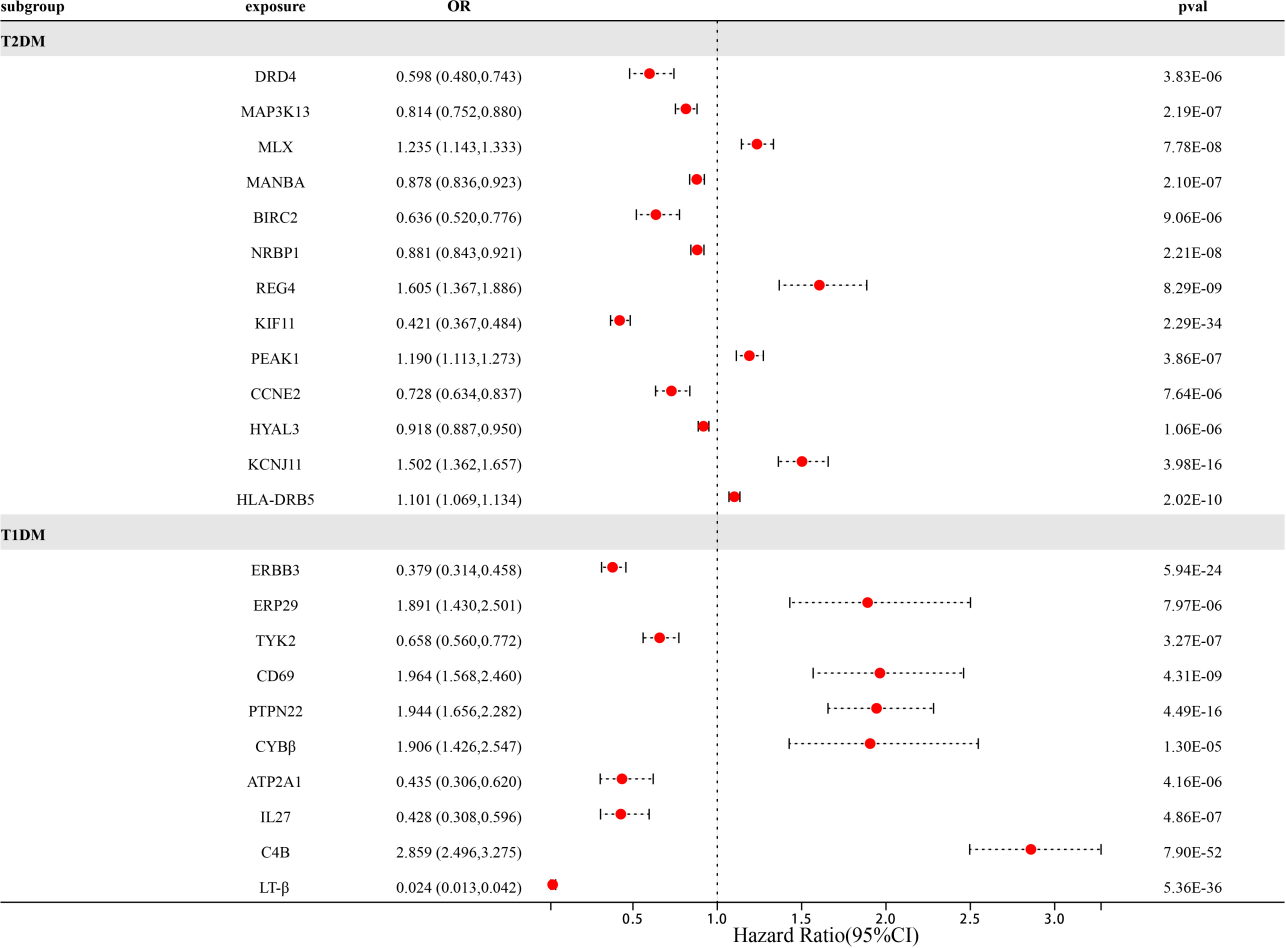
Forest map of 23 potential drug targets for diabetes prevention identified in the dual sample MR phase.

At Bonferroni significance (P<1.90e-05), preliminary MR analysis revealed a total of 23 “druggable” genes associated with T2DM and T1DM (**Figure 2**). Our preliminary results confirm that 13 potential drug targets have significant genetic effects on T2DM (**Table 2**). Specifically, the overexpression of Dopamine Receptor D4 (DRD4) gene, Mitogen-Activated protein kinase 13 (MAP3K13) gene, Mannosidase beta (MANBA) gene, Baculoviral IAP repeat containing 2 (BIRC2) gene, Nuclear receptor Binding protein 1 (NRBP1) gene, Kinesin family member 11 (KIF11) gene, Cyclin E2 (CCNE2) gene and Hyaluronidase 3 (HYAL3) gene is negatively correlated with the risk of developing T2DM (OR values all<1). In contrast, the overexpression of the MAX dimerization protein MLX (MLX) gene, regenerative family member 4 (REG4) gene, pseudopodium-rich atypical kinase 1 (PEAK1) gene, potassium inward rectifying channel subfamily J member 11 (KCNJ11) gene, and major histocompatibility complex class II DRβ5 (HLA-DRB5) gene is positively correlated with the risk of T2DM (OR values all>1). Moreover, our preliminary results also confirm that 10 potential drug targets have significant genetic effects on T1DM (**Table 3**). Specifically, the Erb-b2 receptor tyrosine kinase 3 (ERBB3) gene, Tyrosine kinase 2 (TYK2) gene, ATPase sarcoplasmic/endoplasmic reticulum Ca2+ transporting 1 (ATP2A1) gene, Interleukin-27 (IL-27) geneand Lymphotoxin β (LT- β) gene overexpression is negatively correlated with the risk of developing T1DM (OR values all<1). In contrast, the overexpression of the Endoplasmic reticulum protein 29 (ERP29) gene, CD69 molecule (CD69), protein tyrosine phosphatase non-receptor type 22 (PTPN22) gene, Cytochrome B-245 beta chain (CYBβ) gene, and complement C4B gene is positively correlated with the risk of T1DM (OR values all > 1).

**Table 2:** Significantly correlated MR results of Bonferroni-corrected “druggable” genes with T2DM.

Tissue	Gene-id	Gene	SNP	Method	OR (95% CI)	P-value	PVE	F-statistics
plasma	ENSG00000069696	DRD4	rs1439287	Wald ratio	0.598 (0.480,0.743)	3.83e-06	0.113%	35.48
plasma	ENSG00000073803	MAP3K13	rs7431357	Wald ratio	0.814 (0.752,0.880)	2.19E-07	1.022%	274.76
plasma	ENSG00000108788	MLX	rs60929812	Wald ratio	1.235 (1.143,1.333)	7.78e-08	0.886%	280.21
plasma	ENSG00000109323	MANBA	rs223364	Wald ratio	0.878 (0.836,0.923)	2.10e-07	2.653%	757.86
plasma	ENSG00000110330	BIRC2	rs2013208	Wald ratio	0.636 (0.520,0.776)	9.06e-06	0.114%	36.00
plasma	ENSG00000115216	NRBP1	rs2303370	Wald ratio	0.881 (0.843,0.921)	2.21E-08	2.809%	912.23
plasma	ENSG00000134193	REG4	rs6428842	Wald ratio	1.605 (1.367,1.886)	8.29e-09	0.886%	214.14
plasma	ENSG00000138160	KIF11	rs10882098	Wald ratio	0.421 (0.367,0.484)	2.29E-34	0.284%	87.89
plasma	ENSG00000173517	PEAK1	rs7119	Wald ratio	1.190 (1.113,1.273)	3.86E-07	1.429%	373.98
plasma	ENSG00000175305	CCNE2	rs2515226	Wald ratio	0.728 (0.634,0.837)	7.64E-06	0.277%	87.90
plasma	ENSG00000186792	HYAL3	rs73077175	Wald ratio	0.918 (0.887,0.950)	1.06e-06	6.656%	1690.88
plasma	ENSG00000187486	KCNJ11	rs2074310	Wald ratio	1.502 (1.362,1.657)	3.98E-16	1.154%	166.44
plasma	ENSG00000198502	HLA-DRB5	rs3093990	Wald ratio	1.101 (1.069,1.134)	2.02e-10	7.399%	1980.33

**Table 3:** Significantly correlated MR results of Bonferroni-corrected “druggable” genes with T1DM.

Tissue	Gene-id	Gene	SNP	Method	OR (95% CI)	P-value	PVE	F-statistics
plasma	ENSG00000065361	ERBB3	rs11171739	Wald ratio	0.379 (0.314,0.458)	5.94E-24	1.353%	424.90
plasma	ENSG00000089248	ERP29	rs11066119	Wald ratio	1.891 (1.430,2.501)	7.97E-06	0.889%	236.80
plasma	ENSG00000105397	TYK2	rs1354034 rs12608948 rs149110519 rs34725611	IVW	0.658 (0.560,0.772)	3.27e-07	2.873%	30.08 36.65 56.00 771.59
plasma	ENSG00000110848	CD69	rs117372141 rs1861090	IVW	1.964 (1.568,2.460)	4.31e-09	0.996%	29.78 278.00
plasma	ENSG00000134242	PTPN22	rs2884603	Wald ratio	1.944 (1.656,2.282)	4.49E-16	1.834%	588.75
plasma	ENSG00000165168	CYBβ	rs12478601 rs1375493 rs149007767 rs10980797 rs424971	IVW	1.906 (1.426,2.547)	1.30E-05	2.022%	38.24 30.24 34.27 36.63 42.13
plasma	ENSG00000196296	ATP2A1	rs4788101	Wald ratio	0.435 (0.306,0.620)	4.16E-06	0.383%	121.04
plasma	ENSG00000197272	IL27	rs2925629	Wald ratio	0.428 (0.308,0.596)	4.86e-07	0.791%	151.77
plasma	ENSG00000224389	C4B	rs9264533 rs3132450	IVW	2.859 (2.496,3.275)	7.90e-52	29.44%	328.90 1499.91
plasma	ENSG00000227507	LT-β	rs9267485	Wald ratio	0.024 (0.013,0.042)	5.36E-36	1.195%	69.22

### Sensitivity analysis of 23 potential drug targets in diabetes

In the preliminary analysis of MR, we first identified 23 “druggable” gene targets as potential therapeutic targets for T1DM and T2DM. Including 13 T2DM targets and 10 T1DM targets. To verify the stability of the results, we conducted a series of sensitivity analyses on 23 “druggable” genes (**Tables 4**, **5**). Firstly, we conducted Steiger directionality tests on 23 potential “druggable” gene targets to further ensure directionality. For SNPs with opposite directions, we excluded them to consolidate the stability of the results. Secondly, we conducted a pleiotropy test and found that the P-values for pleiotropy testing were all greater than 0.05 among the 23 “druggable” genes (**Supplementary Table 2**). Thirdly, Bayesian co-localization strongly suggests that in T2DM, CCNE2, HLA-DRB5, KCNJ11, KIF11, MAMBA, MAP3K13, MLX, NRBP1, PEAK1, and REG4 shared the same variant with T2DM (PPH3+PPH4 ≥0.8). Similarly, in T1DM, Bayesian co-localization strongly suggests that ATP2A1, C4B, CD69, ERBB3, IL27, ERP29, PTPN22, TYK2, and LT-β shared the same variant with T1DM (PPH3+PPH4 ≥0.8). It is worth noting that the CYBβ gene serves as a trans-eQTL gene; therefore, Bayesian co-localization analysis was not performed on the CYBβ gene. Fourth, in order to avoid the reverse causal relationship between the 23 “druggable” gene targets and diabetes (T1DM and T2DM), we conducted dual sample reverse MR analysis using T1DM and T2DM as exposure data and 23 eQTLs of “druggable” genes as outcome data, with the IVW method as the main analysis method. We found that the DRD4 (p = 0.021) gene has a bidirectional causal relationship in T2DM, with P values of all other genes > 0.05. In T1DM, we found that the C4B gene (p = 0.000179) had a bidirectional causal relationship, with P values of all other genes > 0.05. This suggests that the DRD4 gene and C4B gene play a bidirectional role in the pathogenesis of T2DM and T1DM, respectively.

**Table 4:** Summary of a series of sensitivity analyses on 13 potential drug targets for T2DM.

Tissue	Gene-id	Gene	Steiger filtering	Bidirectional MR (OR (95% CI)	Bidirectional MR (MR-IVW)	PPH3.abf	PPH4.abf	PPH3+PPH4
plasma	ENSG00000069696	DRD4	TURE (0.028)	0.949 (0.907,0.992)	0.021	1.16E-03	5.24E-03	6.40E-03
plasma	ENSG00000073803	MAP3K13	TURE (2.29E-28)	1.005 (0.952,1.061)	0.838	2.55E-02	9.74E-01	9.995E-01
plasma	ENSG00000108788	MLX	TURE (4.74E-26)	0.999 (0.932,1.070)	0.979	4.91E-01	5.09E-01	1.00
plasma	ENSG00000109323	MANBA	TURE (1.59E-88)	0.938 (0.833,1.056)	0.294	4.81E-01	5.18E-01	9.99E-01
plasma	ENSG00000110330	BIRC2	TURE (0.019)	1.038 (0.993,1.085)	0.096	3.12E-03	8.08E-02	0.0839E-02
plasma	ENSG00000115216	NRBP1	TURE (1.27E-100)	1.015 (0.833,1.122)	0.659	1.00E+00	2.01E-08	1.00
plasma	ENSG00000134193	REG4	TURE (1.38E-30)	0.949 (0.907,1.072)	0.594	1.00E+00	6.24E-07	1.00
plasma	ENSG00000138160	KIF11	TURE (0.50)	0.979 (0.908,1.056)	0.593	1.17E-01	8.83E-01	1.00
plasma	ENSG00000173517	PEAK1	TURE (1.37E-41)	0.991 (0.924,1.063)	0.817	1.00E+00	7.63E-07	1.00
plasma	ENSG00000175305	CCNE2	TURE (4.36E-07)	0.994 (0.925,1.069)	0.884	5.47E-01	4.51E-01	9.98E-01
plasma	ENSG00000186792	HYAL3	TURE (7.24E-226)	1.009 (0.956,1.065)	0.740	2.41E-03	1.13E-02	1.37E-02
plasma	ENSG00000187486	KCNJ11	TURE (4.38E-16)	1.061 (0.969,1.162)	0.199	1.04E-01	8.96E-01	1.00
plasma	ENSG00000198502	HLA-DRB5	TURE (8.39E-251)	0.818 (0.569,1.178)	0.281	1.00E+00	8.32E-06	1.00

**Table 5:** Summary of a series of sensitivity analyses on 11 potential drug targets for T2DM.

Tissue	Gene-id	Gene	Steiger filtering	Bidirectional MR (OR (95% CI)	Bidirectional MR (MR-IVW)	PPH3.abf	PPH4.abf	PPH3+PPH4
plasma	ENSG00000065361	ERBB3	TURE (5.38E-10)	0.969 (0.882,1.065)	0.524	2.19E-02	9.78E-01	9.99E-01
plasma	ENSG00000089248	ERP29	TURE (6.67E-14)	0.996 (0.981,1.012)	0.692	1.00E+00	1.01E-11	1.00
plasma	ENSG00000105397	TYK2	TURE (0.090–1.29E-49)	0.982 (0.929,1.039)	0.549	3.10E-02	9.68E-01	9.99E-01
plasma	ENSG00000110848	CD69	TURE (0.020–3.67E-12)	1.024 (0.951,1.103)	0.517	1.43E-01	8.57E-01	1.00
plasma	ENSG00000134242	PTPN22	TURE (1.76E-23)	1.040 (0.971,1.114)	0.253	1.00E+00	1.68E-58	1.00
plasma	ENSG00000165168	CYBβ	TURE (1.07E-03–1.10E-06)	1.000 (0.979,1.020)	0.995	–	–	–
plasma	ENSG00000196296	ATP2A1	TURE (1.12E-04)	0.997 (0.975,1.019)	0.813	8.28E-02	9.13E-01	9.95E-01
plasma	ENSG00000197272	IL27	TURE (3.62E-04–4.65E-08)	1.003 (0.964,1.043)	0.864	5.22E-01	4.78E-01	1.00
plasma	ENSG00000224389	C4B	TURE (6.55E-36–3.53E-137)	1.090 (1.042,1.141)	1.79E-04	1.00E+00	5.39E-31	1.00
plasma	ENSG00000227507	LT-β	TURE (0.038)	1.014 (0.977,1.053)	0.449	1.00E+00	2.55E-12	1.00

### External validation of potential drug targets for diabetes

**Figure 3:**
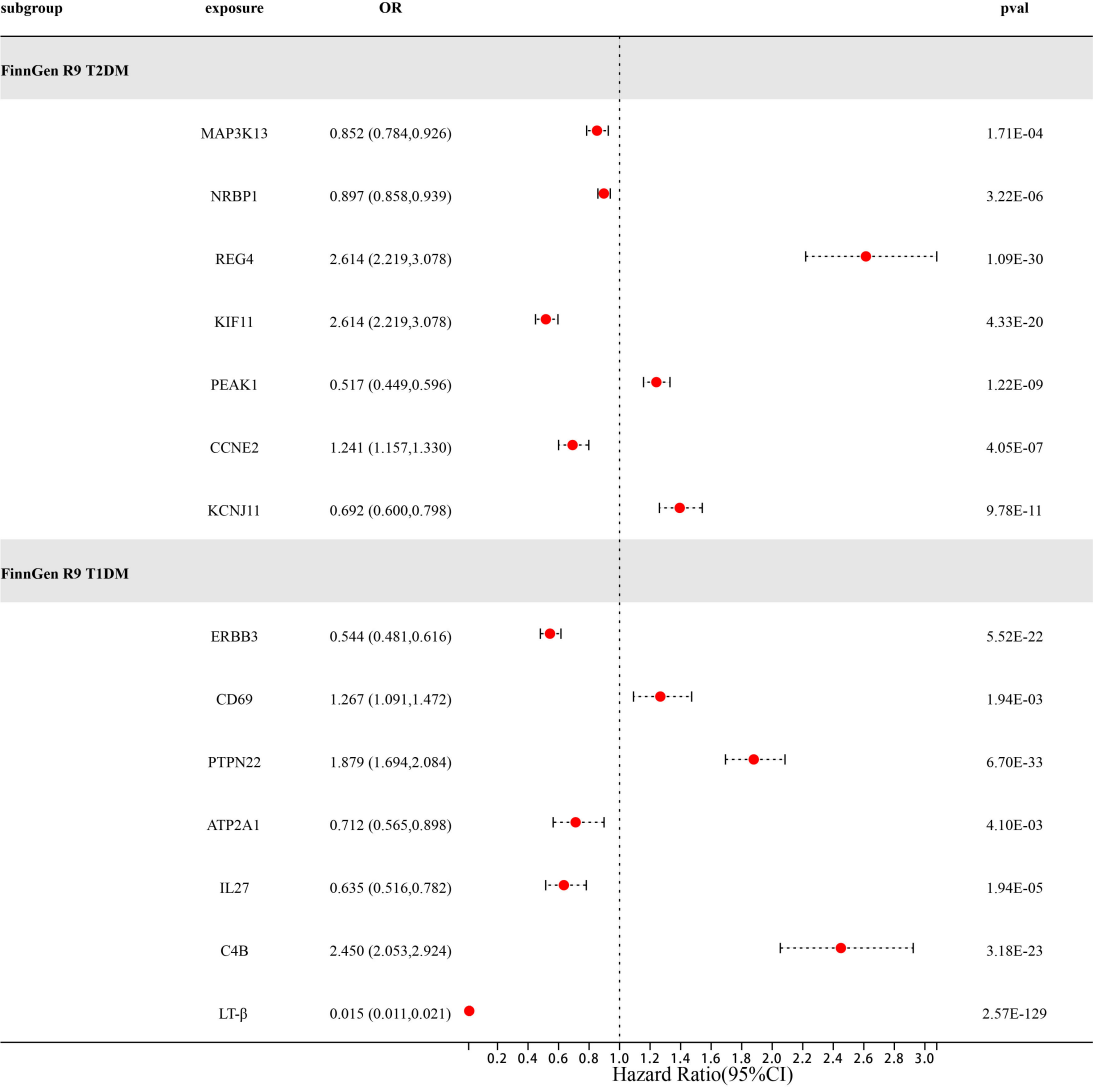
Forest diagram of 14 potential drug targets confirmed through external validation of FinnGen R9T1DM and T2DM.

After a series of quality control steps such as sensitivity, co-localization, and reverse MR analysis, we deem that 21 gene targets have the most reliable MR evidence for T1DM and T2DM risk. C4B gene has a bidirectional causal relationship in T1DM risk. However, due to differences in genetic effects between different datasets. However, due to differences in genetic effects between different datasets, Therefore, we use the same-variant and significant-variant strategies in different datasets to replicate the primary findings and further determine the stability of our results (**Table 6**; **Supplementary Table 3**). We selected T1DM, T2DM, and their six related complications from the FinnGen R9 study as the external validation set. In external validation, we found that seven target genes (P<0.0038) were replicated in T2DM, namely MAP3K13, NRBP1, REG4, KIF11, PEAK1, CCNE2, and KCNJ11 (**Figure 3**). Among them, REG4 has a strong correlation with T2DM and can be replicated in all six complications of T2DM; KIF11 is replicated in four complications of T2DM; and KCNJ11 is replicated in three complications of T2DM (**Supplementary Table 4**). Same, Not only that, we also found a strong correlation between C4B, LT-β, and T1DM. C4B and LT-β were replicated in six complications of T1DM; ERBB3 and PTPN22 were replicated in five complications of T1DM; and IL27 was replicated in five complications of T1DM (**Supplementary Table 5**).

**Table 6:** The main results of external validation after Bonferroni correction.

Tissue	Gene-id	Gene	Outcome	Method	OR (95% CI)	P-value
plasma	ENSG00000073803	MAP3K13	T2DM	Wald ratio	0.852 (0.784,0.926)	1.71e-04
plasma	ENSG00000115216	NRBP1	T2DM	Wald ratio	0.897 (0.858,0.939)	3.22e-06
plasma	ENSG00000134193	REG4	T2DM	Wald ratio	2.614 (2.219,3.078)	1.09e-30
plasma	ENSG00000138160	KIF11	T2DM	Wald ratio	0.517 (0.449,0.596)	4.33e-20
plasma	ENSG00000173517	PEAK1	T2DM	Wald ratio	1.241 (1.157,1.330)	1.22e-09
plasma	ENSG00000175305	CCNE2	T2DM	Wald ratio	0.692 (0.600,0.798)	4.05e-07
plasma	ENSG00000187486	KCNJ11	T2DM	Wald ratio	1.394 (1.261,1.542)	9.78e-11
plasma	ENSG00000065361	ERBB3	T1DM	Wald ratio	0.544 (0.481,0.616)	5.52e-22
plasma	ENSG00000110848	CD69	T1DM	IVW	1.267 (1.091,1.472)	1.94e-03
plasma	ENSG00000134242	PTPN22	T1DM	Wald ratio	1.879 (1.694,2.084)	6.70e-33
plasma	ENSG00000196296	ATP2A1	T1DM	Wald ratio	0.712 (0.565,0.898)	4.10e-03
plasma	ENSG00000197272	IL27	T1DM	Wald ratio	0.635 (0.516,0.782)	1.94e-05
plasma	ENSG00000224389	C4B	T1DM	IVW	2.450 (2.053,2.924)	3.18e-23
plasma	ENSG00000227507	LT-β	T1DM	Wald ratio	0.015 (0.011,0.021)	2.57e-129

### Association between potential drug targets and biomarkers of diabetes

The primary pathophysiological features of T2MD are impaired insulin secretion and insulin resistance, with insulin resistance being the principal cause of T2MD (22). Certain biomarkers, lifestyle, environmental, dietary, and socio-psychological factors contribute to the risk of insulin resistance and are also non-genetic risk factors for T2DM (23). Previous studies have determined that certain clinical characteristics (age, body mass index (BMI), waist-to-hip ratio, and hypertension) are associated with the risk of T2DM and a series of metabolic characteristics related to diabetes (24). There is a causal relationship between BMI and a 26% increase in T2DM risk, and a causal relationship between BMI adjusted waist to hip ratio and a 38% increase in T2DM risk (25). The coexistence of T2DM and hypertension significantly increases the risk of cardiovascular disease, end-stage renal disease, and death (26). Secondly, in a large-scale, 10-year prospective study of T2DM patients (the METSIM study), it was observed that vitamin D, branched-chain amino acids, low-density cholesterol, triglycerides, proinsulin levels, fatty acids, glycerol, mannose, glycoprotein acetyl (GlycA), and acetyl acetate are associated with an increased risk of T2DM and also serve as T2DM biomarkers (27, 28). Mannose is a hexose required for glycoprotein synthesis, which is significantly elevated in insulin-resistant subjects (29). Acetoacetate (ketone body) is an important marker of T2DM ketoacidosis (30). GlycA is associated with chronic inflammation of the pancreas and serves as a marker for predicting impaired insulin secretion (31). Insulin regulates the concentration of glycerol, triglycerides, and fatty acids in the serum by inhibiting fat breakdown. Therefore, the concentration of lipids in plasma also serves as a marker for predicting T2DM (32). Vitamin D may regulate insulin resistance and the pancreas β Cellular function plays a role in the pathogenesis of T2DM (33). The ratio of apolipoprotein to low-density cholesterol (LDL) is a predictor of worsening blood glucose and the incidence rate of T2DM (34).

In order to explore the potential role of identified drug targets in the pathogenesis of T2DM, we used the previously identified 7 potential “druggable” targets eQTL for T2DM as exposure data and 10 T2DM biomarkers (vitamin D, branched-chain amino acids, low-density cholesterol, triglycerides, proinsulin levels, fatty acids, glycerol, mannose, glycoprotein acetyl, and acetoacetate) and 3 clinical features (BMI, hypertension, and waist-to-hip ratio) as outcome data to further evaluate the genetic effects of potential “druggable” targets and T2DM biomarkers (**Supplementary Table 6**). We found that under P-values <0.05 (P<0.05), the level of vitamin D is only related to the KCNJ11 gene. The level of branched-chain amino acids is related to the NRBP1, KIF11, and CCNE2 genes. The level of LDL is related to the REG4, KIF11, and KCNJ11 genes. The level of triglycerides is related to the NRBP1, REG4, KIF11, and CCNE2 genes. The levels of proinsulin, mannose, and glycerol are only related to the NRBP1 gene. The level of total fatty acid is related to the NRBP1, MAP3K13, REG4, and KIF11 genes. The level of glycoprotein acetyl is related to the NRBP1, MAP3K13, REG4, KIF11, and PEAK1 genes. The level of acetoacetate is related to the REG4 and PEAK1 genes. The risk of obesity (BMI) is related to the NRBP1, KIF11, and KCNJ11 genes. The risk of hypertension is related to the NRBP1, REG4, CCNE2, and KCNJ11 genes. The waist-to-hip ratio is related to the MAP3K13, NRBP1, and KIF11 genes. (**Supplementary Table 7**).

## Discussion

To our knowledge, this work is the first to combine eQTL data from “druggable” genomics. Through the use of double-sample MR, Bayesian co-localization, and external validation of different data sets, we explored the potential pathophysiological mechanisms of T1DM and T2DM and specifically sought to identify new drug targets for diabetes treatment. In this study, we finally found 14 potential drug targets to prevent diabetes, including 7 “druggable” genes that may affect the expression of T1DM results and 7 “druggable” genes that may affect the expression of T2DM results.

Mitogen-activated protein kinase kinase 13 (MAP3K13), also known as leucine zipper kinase (LZK), has a high degree of homology with leucine zipper kinase (DLK). The common feature of both is the presence of a double leucine zipper (LZ) domain after the kinase domain, which can activate JNK and, to some extent, activate p38 MAPK to exert biological activity (35). The human DLK kinase domain consists of 127–375 amino acid residues, including four characteristic domains: the catalytic domain, the double leucine zipper domain, the glycine serine proline rich domain, and the glycine proline rich domain (36). In many previous studies, it has been confirmed that DLK is related to the occurrence of type 2 diabetes, and DLK is widely present in pancreatic islets. In the experiment of E. Oetjen et al., it was found that the overexpression of DLK in pancreatic β cells decreased the transcriptional activity of CBP/CERB, leading to decreased insulin secretion and the occurrence of diabetes (37). In the study by M.J. Stahnke et al., it was also found that overexpression of DLK not only inhibits the transcriptional activity of the human insulin gene promoter but also leads to a decrease in the function and quality of pancreatic islet β cells. Not only that, they also found that overexpression of DLK can induce the degradation of the insulin gene transcription factor v-Maf musculoaponeurotic fibrosarcoma oncogene family, protein A (MafA) (38). Mafa is an alkaline leucine zipper family transcription factor that can activate the expression of insulin in β-cells with PDX1 and NEUROD1. Therefore, MafA is essential for maintaining both adult β cells and β cellular function (39). Moreover, inhibiting DLK kinase may protect β cells from the influence of β cytotoxic pre-diabetes signals and prevent the development of diabetes (40). Therefore, MAP3K13/DLK is expected to become a new target for the treatment of T2DM.

Potassium inwardly rectifying channel subfamily J member 11 (KCNJ11) is a target most closely related to T2DM in our study and also an achievement based on the precise treatment of monogenic insulin deficiency and insulin resistance diabetes in the new century (41). The potassium inward rectifying 6.2 subunit (Kir6.2) of the ATP-sensitive potassium (K (ATP)) channel encoded by the KCNJ11 gene is a therapeutic target for sulfonylurea drugs and plays an important role in regulating glucose homeostasis. At present, sulfonylurea drugs targeting KCNJ11 (glibenclamide, gliclazide) are widely used as first-line treatment drugs for T2DM in clinical treatment (42).

Regenerating family member 4 (REG4) is a member of the calcium-dependent (C-type) lectin superfamily, mainly expressed in gastrointestinal tissues, including the colon, small intestine, stomach, and pancreas (43). Previous studies have found that REG4 plays an important role in pancreatic cell and duct regeneration. In Hu et al.’s experiment, it was found that the expression levels of Reg4 mRNA and protein were significantly increased during acute pancreatitis (AP). In addition, Reg4 upregulates the expression of Bcl-2 or Bcl-xL by activating the EGFR/Akt pathway, which can prevent arginine-induced acinar cell necrosis both *in vivo* and *in vitro* (44). This was also confirmed in another study (45). Moreover, in the animal experiment constructed by Wang et al. using rats, it was found that Reg4 may mediate intestinal fatty acid extraction and absorption through AMPK. The expression of Reg4 can reduce intestinal fat absorption to protect mice from high-fat diet-induced hepatic steatosis, increased fat accumulation, and insulin resistance (46). It is worth noting that although Reg4 plays a certain role in pancreatic regeneration, it also plays an important role in the occurrence, development, and invasion of pancreatic cancer as a serum marker of cancer (47).

Kinesin family member 11 (KIF11) belongs to the kinesin-5 family and is a protein-coding gene that plays an important role in cell mitosis, cell cycle, and differentiation. Previous studies have found that KIF11 is closely associated with familial exudative vitreoretinopathy, lymphedema, intellectual disability, chorioretinopathy, and an increased risk of T2DM (48, 49). Four single nucleotide polymorphisms (SNPs) of the KIF11 gene identified in the GWAS catalogue are associated with diabetes, including RS2153827, RS6583826, and RS7087591, which are all associated with the risk of T2DM, while RS7096101 is associated with insulin level. Moreover, KIF11 did not show significant racial differences in the risk of T2DM. KIF11 was found as a genetic variation associated with T2DM risk in large GWAS data from multiple ethnic cohorts, including Asian (Chinese, Japanese), European, and Southeast Asian populations (50–52). KIF11 has also been confirmed to be related to type 2 diabetes retinopathy, which is consistent with our external verification results (53). In summary, KIF11 is also expected to become a potential target for the treatment of T2DM.

Cyclin E2 (CCNE2) belongs to the highly conserved cyclin family and is a protein-coding gene that functions as a regulator of cell cycle-dependent protein kinase (CDK). The cyclin box domain in CCNE2 is an essential structural motif for the formation of cyclin CDK complexes. CCNE2 binds to CDK2 in a functional kinase complex with catalytic activity and plays a role in the G1/S transition of the cell cycle, reaching its peak expression in the G1-S phase. Plays an important role in cell proliferation and regeneration (54, 55). Therefore, the function of CCNE2 affects the cell cycle kinase CDK2 and the progression of the cell cycle. However, disruption of CDK2 and the cell cycle can lead to dysfunction of pancreatic β cells and a decrease in β cell mass, accelerating the progression of T2DM. In Yoon Kim et al.’s experiment, it was found that the absence of CDK2 can impair the function of adult β cells, affect the quality of pancreatic β cells, and lead to insulin secretion defects (56). This phenomenon was also confirmed in Jiang et al.’s experiment (57). In addition, CDK2 agonists may help to restore β cell function and restart β cell proliferation to combat β cell failure in diabetes patients. Therefore, CCNE2 is expected to become a potential therapeutic target for T2DM patients.

PEAK1 and NRBP1 are the two genes that we found to have the least correlation with reported T2DM. PEAK1 encodes a non-receptor tyrosine kinase, which is a member of the novel kinase family three (NFK3) family. This gene is associated with the actin cytoskeleton and adhesive plaques and plays a role in regulating cell migration, proliferation, and cancer metastasis (58). At present, the research on PEAK1 is more focused on its role in tumor metastasis and invasion, so the research on PEAK1 and diabetes, especially T2DM, needs further discussion. There are few reports about NRBP1 and diabetes. At present, studies believe that the expression of the NRBP1 gene is related to apo CIII sialylation and hypertriglyceridemia. However, apo CIII is involved in triglyceride-rich lipoprotein metabolism and is associated with β cell damage, insulin resistance, and cardiovascular disease (59).

T1DM is an autoimmune disease caused by complex interactions between multiple susceptible genes, environmental factors, and the immune system. Its characteristic is T-cell-mediated self-destruction of pancreatic β cells, leading to an absolute lack of insulin secretion (60). At present, the treatment of T1DM often relies on exogenous insulin. However, in our study, we found seven genes closely related to the pathophysiology of T1DM. They are ERBB3, C4B, CD69, PTPN22, IL27, ATP2A1, and LT -β respectively. ERBB3, CD69, PTPN22, and IL27 have a highly significant correlation with T1DM. These four genes are four of more than 50 loci significantly related to human non-HLA T1DM identified by the Genetic Alliance for T1DM (61). ERBB3 is located on chromosome 12q13, also known as Her3 (human epidermal growth factor receptor 3), and is a member of the epidermal growth factor receptor (EGFR) family of receptor tyrosine kinases (62). A study has found that the sites rs11171739 and rs2292239 associated with ERBB3 affect the occurrence and development of T1DM (63). Firstly, ERBB3 is considered a novel regulator of pancreatic β cell apoptosis, and downregulation of ERBB3 can reduce basal cell and cytokine-induced cell apoptosis. Secondly, ERBB3 can activate various downstream signaling pathways, such as phosphoinositol 3-kinase (PI3K), nuclear factor κB (NF-κB), and extracellular signal-regulated kinase (ERK), among which the PI3K pathway is an important survival pathway for pancreatic β cells (64, 65). There are also studies indicating that ERBB3 can regulate β-cellulose (BTC) to promote β cell regeneration (66). IL27 is a heterodimeric cytokine that plays a role in innate immunity, regulating helper T cell development, inhibiting T cell proliferation, and stimulating cytotoxic T cell activity. It has multiple effects on innate immune cells (67). IL27 can target CD4 helper T cells and promote the differentiation of type 1 effector cells (TH1) and type 2 effector cells (TH2). Previous studies have found that SNP rs4788084, located within 2kb upstream of IL27, serves as a T1D-related risk allele (61, 68). IL-27 plays a protective role in the development of T1DM and has anti-inflammatory properties by regulating T cell polarization and cytokine levels. Overexpression of IL-27 can reduce pancreatic blood glucose levels, immune cell infiltration, and the expression of the anti-inflammatory cytokine IL-1β mRNA, thereby alleviating inflammation of the pancreas (69). Secondly, IL-27 directly affects the differentiation and effector function of CD4 and CD8 T cells in the pancreas, enhancing the expression of T-bet and IFN-γ in the pancreas (70). IL-27 can also directly change the balance between islet regulatory T cells (Tregs) and helper T cells 1 (Th1), thereby regulating the diabetic activity of CD8 T cells (71).

The CD69 gene encodes a member of the type II transmembrane receptor calcium-dependent lectin superfamily and is expressed on various white blood cells, including newly activated lymphocytes, certain subtypes of memory T cells, regulatory T cells (Tregs), and natural killer (NK) T cells (72). CD69 is a marker of early lymphocyte activation, highly upregulated in T1DM lymphocytes. CD69 exerts its immune regulatory function by controlling the balance between Th/Treg cell differentiation and enhancing Treg inhibitory activity, thereby regulating immune tolerance and lymphocyte infiltration in pancreatic islet cells (73). The protein encoded by the protein tyrosine phosphatase non-receptor 22 (PTPN22) gene is a lymphoid-specific intracellular phosphatase that participates in regulating the function of the CBL protein in the T cell receptor signaling pathway by binding to the molecular adaptor protein CBL. Previous studies have shown that the allele variant PTPN22 R620W of the PTPN22 gene is the strongest non-HLA genetic risk factor for the development of T1DM. However, the pathogenesis of PTPN22 in T1DM is complex, and current research generally suggests that the expression of PTPN22 may play a role in β cell apoptosis and affect β cell function (74).

C4B is located in the major histocompatibility complex (MHC) class III region on chromosome 6, encoding the basic form of complement factor 4, and is part of the classical activation pathway along with the C4A gene. The C4B-related pathways include complement cascade response and regulation of insulin-like growth factor (IGF) transport and insulin-like growth factor binding protein (IGFBP) uptake. The immune deficiency caused by complement deficiency in the classical pathway plays an important role (75). This also indicates that the occurrence of T1DM is closely related to the complement pathway. The complement components C3 and C4 are highly expressed in the pancreas (including islets), and the copy number of the C4B gene or higher C4B protein concentration can affect the ability of endogenous insulin or C-peptide (76). In addition, studies have found that during insular encephalitis, the complement cascade reaction is activated, and complement factors deposit into the pancreas, thereby damaging the pancreas and pancreatic islet cells. Moreover, the increase in serum complement C4B level is also related to diabetes nephropathy (77). ATPase sarcoplasmic/endoplasmic reticulum Ca2+transporter 1 (ATP2A1) is a protein-coding gene that encodes a SERCA Ca2+ ATPase enzyme, which plays an important role in maintaining low concentrations of Ca2+ions in the cytoplasm (78). However, intracellular Ca2+ is an important coordinating agent in various aspects of cellular physiology, and changes in cellular Ca2+ dynamics help regulate normal and pathological signal transduction that controls cell growth and survival. The disturbance of Ca2+ concentration is closely related to ER stress and mitochondrial dysfunction, which are the two major defects leading to T1DM and T2DM (79). The changes in SERCA function, endoplasmic reticulum stress (ER), and Ca2+ cycle are the basis of the mechanism of pancreatic β cell apoptosis. A study has found that SERCA2 deficiency can lead to an increase in basal cytoplasmic Ca2+ levels, directly leading to a decrease in insulin secretion, a decrease in β cell mass, and an increase in β cell ER stress and death (80, 81). Lymphotoxin-β (LT-β) is a protein-coding gene that functions as a biological cousin of tumor necrosis factor α (TNFα) by binding to the lymphotoxin p receptor (LTβR) and participating in the normal development of lymphoid tissue. LT-β participates in the pathogenesis of T1D by promoting the formation of the tertiary lymphoid organ (TLO) around the pancreas and also enhances the autoimmune response by regulating the synergistic effect of multiple cytokines and inflammatory mediators, inducing insulinitis and peripancreatic inflammation, leading to β cell destruction (82).

Our research provides several significant advantages. Firstly, we used multiple sample datasets to validate our results, and the larger the sample size, the higher the ability to detect eQTL. Secondly, we used multiple methods such as multivariate MR analysis, MR Steiger filtering, reverse MR, co-localization analysis, and pleiotropy testing to validate our results, further ensuring the reliability of our results. As is well known, the success rate of drugs from phase 1 trials to approval is very low, requiring significant investment of manpower, material resources, and financial resources. Although MR analysis cannot replace *in vitro* and *in vivo* preclinical evaluations of drug targets, combined genomics methods can serve as an auxiliary tool for drug development, with the potential to better prioritize drug targets entering clinical trials. Therefore, this may have a significant impact on drug development costs. However, we have to admit that our research also has some limitations. First, the use of genetic variation to study and determine the effect of potential therapeutic drugs for diabetes is limited because the pathogenesis of diabetes is complex and involves a multi-gene, multi-factor, and multi-target pathogenic pathway. Therefore, drug MR analysis helps to determine the direction of causal relationships rather than quantifying the degree of correlation. Secondly, the number of IVs in eQTL MR is limited, with most not exceeding three SNPs, which limits the credibility of MR results. Thirdly, although we conducted detailed heterogeneity and pleiotropy tests, we cannot completely rule out the influence of horizontal pleiotropy. Fourthly, as our research findings mainly relate to individuals of European ancestry, these findings may not necessarily apply to other racial groups. Finally, despite the large sample size of our study, the several genetic tools used for the results are affected to varying degrees by low statistical power and incomplete phenotype definitions, which may render most of the explored association studies ineffective. Therefore, our results still need to be validated in larger queues. Even validated in clinical trials.

## Data availability statement

The raw data supporting the conclusions of this article will be made available by the authors, without undue reservation.

## Ethics statement

Ethical approval was not required for the study involving humans in accordance with the local legislation and institutional requirements. Written informed consent to participate in this study was not required from the participants or the participants’ legal guardians/next of kin in accordance with the national legislation and the institutional requirements.

## Author contributions

HL: Conceptualization, Data curation, Formal analysis, Writing – original draft. WL: Software, Supervision, Writing – review & editing. DL: Writing – review & editing. LY: Writing – review & editing. YX: Writing – review & editing. PS: Writing – review & editing. LW: Writing – review & editing. ZZ: Funding acquisition, Project administration, Supervision, Writing – original draft, Writing – review & editing.
